# Case report: Fatal neonatal sepsis associated with *Escherichia fergusonii* infection in a common bottlenose dolphin (*Tursiops truncatus*)

**DOI:** 10.3389/fvets.2023.1242599

**Published:** 2023-09-26

**Authors:** Su-Min Baek, Seoung-Woo Lee, Tae-Un Kim, Ji Hyung Kim, Young-Jin Lee, Jae-Hyuk Yim, Woo Jun Kim, Seong-Kyoon Choi, Jee Eun Han, Kyu-Shik Jeong, Jin-Kyu Park

**Affiliations:** ^1^Department of Veterinary Pathology, College of Veterinary Medicine, Kyungpook National University, Daegu, Republic of Korea; ^2^Cardiovascular Research Institute, Kyungpook National University, Daegu, Republic of Korea; ^3^Core Protein Resources Center, Daegu-Gyeongbuk Institute of Science and Technology (DGIST), Daegu, Republic of Korea; ^4^Infectious Disease Research Center, Korea Research Institute of Bioscience and Biotechnology, Daejeon, Republic of Korea; ^5^Department of Food Science and Biotechnology, Gachon University, Seongnam, Republic of Korea; ^6^College of Veterinary Medicine, Kyungpook National University, Daegu, Republic of Korea

**Keywords:** sepsis, *Escherichia fergusonii*, dolphin, neonate, cetaceans

## Abstract

A 25-day-old male common bottlenose dolphin (*Tursiops truncatus*) died suddenly while swimming at a dolphinarium. The gross examination revealed ulceration on the dorsal and pectoral fins and rostrum. Severe congestion, hemorrhage, and edema were observed in the gastrointestinal tract, liver, mesenteric lymph nodes, lungs, and kidneys. Fibrinosuppurative arthritis of the atlantooccipital joint and extension of fibrin into the spinal canal caused compression of the spinal cord. Histopathological examination revealed tracheitis, fibrinosuppurative bronchopneumonia and enteritis. In the central nervous system, meningeal vessel congestion in the brain, and intraparenchymal hemorrhages with neurodegeneration were observed in the spinal cord. Based on the histopathological findings, representative samples, including lung, liver, mesenteric lymph node, blood obtained from the jugular vein, and fluid sample of the ascites, were inoculated on tryptic soy agar and blood agar for routine bacterial isolation. Each isolated bacterial colony was streaked aseptically onto tryptic soy agar and blood agar for pure culture. After then, polymerase chain reaction (PCR) was performed for further identification of pathogenic microorganisms. PCR identified *Escherichia fergusonii*, *Shewanella haliotis*, *Enterococcus faecalis*, and *Staphylococcus schleiferi*. *E. fergusonii* was considered the primary etiologic agent in this case since it was the only species identified in all representative samples. The cause of death in this animal was *E. fergusonii* sepsis. To the best of our knowledge, this is the first case of neonatal sepsis associated with *E. fergusonii* infection in a dolphin, and suggests *E. fergusonii* as an opportunistic pathogen associated with sepsis in dolphins.

## Introduction

Sepsis is a life-threatening condition caused by serious bacterial infections accompanied by a systemic inflammatory response, fever, and organ failure (1). Sepsis is one of the main causes of high neonatal mortality in veterinary medicine (2–4). Several bacterial agents have been associated with septicemia in cetaceans. *Brucella* sp., *Erysipelothrix rhusiopathiae*, *Staphylococcus aureus*, and *Streptococcus* Group D have been reported to cause mortality, tissue abnormality accompanied by clinical signs including pneumonia, vertebral osteomyelitis, and septicemia, and histopathological and hematological signs such as inflammation in the liver and lungs and neutrophilic leukocytosis (5). Several Gram-negative bacteria *Aeromonas* sp., *Edwardsiella* sp., *Pasteurella* sp., *Salmonella* sp., *Vibrio* sp., and Gram-positive bacteria *Corynebacterium* sp., and *Nocardia* sp. have been reported to cause sepsis in cetacean as well (6). Especially, in dolphins, sepsis has been reported due to several etiological agents such as *Erysipelothrix rhusiopathiae* (7), *Streptococcus iniae* (8), and *Escherichia coli* (9). Another bacterial pathogen, *Brucella ceti* has been known to cause sepsis, meningoencephalitis, arthritis, abscess in a variety of organs, inflammation in reproductive system, abortions, placentitis, and pneumonia acquired *in utero* in cetaceans (10). *Escherichia fergusonii* is a rare opportunistic pathogen that has demonstrated multidrug resistance (11, 12). It has been isolated from foods, water, blood, urine, wounds, and intestinal contents of humans, and fecal sample of animals including goat, sheep, horse, poultry, cattle, and pigs (11, 13, 14). This species causes severe health problems in both humans and animals. It has been associated with open wound infection, bacteremia, and urinary tract infection in humans (11, 12) as well as diarrhea, respiratory disease, mastitis, meningitis, abortion, and septicemia in animals (15). Despite its clinical significance, very few case of *E. fergusonii* infection has previously reported in marine mammals.

In particular, cases of sepsis caused by *E. fergusonii* are extremely rare, no cases have been reported in dolphins to date. Herein we report the first case of *E. fergusonii* sepsis in a bottlenose dolphin calf born in captivity.

## Case description

A male common bottlenose dolphin (age: 25 days, body weight: 20 kg, and total length: 110 cm) from a local dolphinarium was necropsied. In the dolphinarium, the pools were filled with 50 tons of filtered sea water every day. There were four dolphins living in the pools, but each section was separated, and only the calf and the mother used the same section of the pools. Water quality was checked three times a day and the water temperature was maintained between 21 and 25°C. Respiration, feeding, and defecation rates of the dolphins were checked all day and their behaviors were monitored with CCTVs and underwater cameras. There were no health problems observed in the mother and other dolphins that were in the same water condition. The calf had showed occasional trembling of the fluke, but he had no relevant medical history. The calf was cared by the mother after birth and there was no problem with gestation or lactation. Twenty five days after birth, the dolphin suddenly died while swimming, with no obvious clinical symptoms prior to death.

Complete necropsy was performed 24 h after death, and the body had been stored at 4°C before necropsy. Blood sample was collected from the jugular vein right after the skin incision, but before the dissection of thoracic and abdominal cavities to avoid postmortem contamination with gut contents, urine, or other body fluids. Jugular vein was punctured with sterile syringe with 23G needle. Collected blood samples were immediately incubated in Amies transport medium (COPAN Diagnostics, Murrieta, CA, United States) and BD Vacutainer™ Plastic Blood Collection Tubes (Becton Dickinson, Franklin Lakes, NJ, United States). Representative organ samples were fixed in 10% neutral formalin for further histopathological analysis. After overnight fixation, the tissues were additionally trimmed and fixed in 10% neutral formalin, for 1 day more to improve fixation. After then, fixed tissues were processed routinely, and embedded in paraffin. These paraffin-embedded tissue blocks were cut to obtain 4-μm-thick sections, which were subjected to hematoxylin and eosin (H&E), Gram staining for further analysis.

Bacteriological isolation and molecular analysis were performed as previously described (16). Representative samples collected with Amies transport medium (COPAN Diagnostics) from the lungs and liver, blood obtained from the jugular vein, and fluid sample of the ascites were inoculated on tryptic soy agar (Hanil Komed, Gyeonggi, Republic of Korea) and blood agar (BD Difco, Sparks, MD, United States) for routine bacterial isolation. Each isolated bacterial colony was streaked aseptically onto tryptic soy agar and blood agar for pure culture. Polymerase chain reaction (PCR) was performed for further identification of the bacterial species. Isolated bacterial colonies were subject to amplification by PCR using the universal primers 27F (5′-AGA GTT TGA TCC TGG CTC AG-3′) and 1492R (5′-GGT TAC CTT GTT ACG ACT T-3′) as described previously (16). After the amplification, the amplicons were purified and sequenced with 785F (5′-GGATTAGATACCCTGGTA-3′) and 907R (5′-CCGTCAATTC MTTTRAGTTT-3′) by using ABI 3730xl System (Macrogen Inc., Seoul, Republic of Korea). The sequences of the colonies were compared and the related taxa were screened against the NCBI rRNA database^1^ to identify the bacterial strains. The sequence of *E. fergusonii* detected in the present study was deposited in GenBank with accession number OR404993.^2^

**Figure 1:**
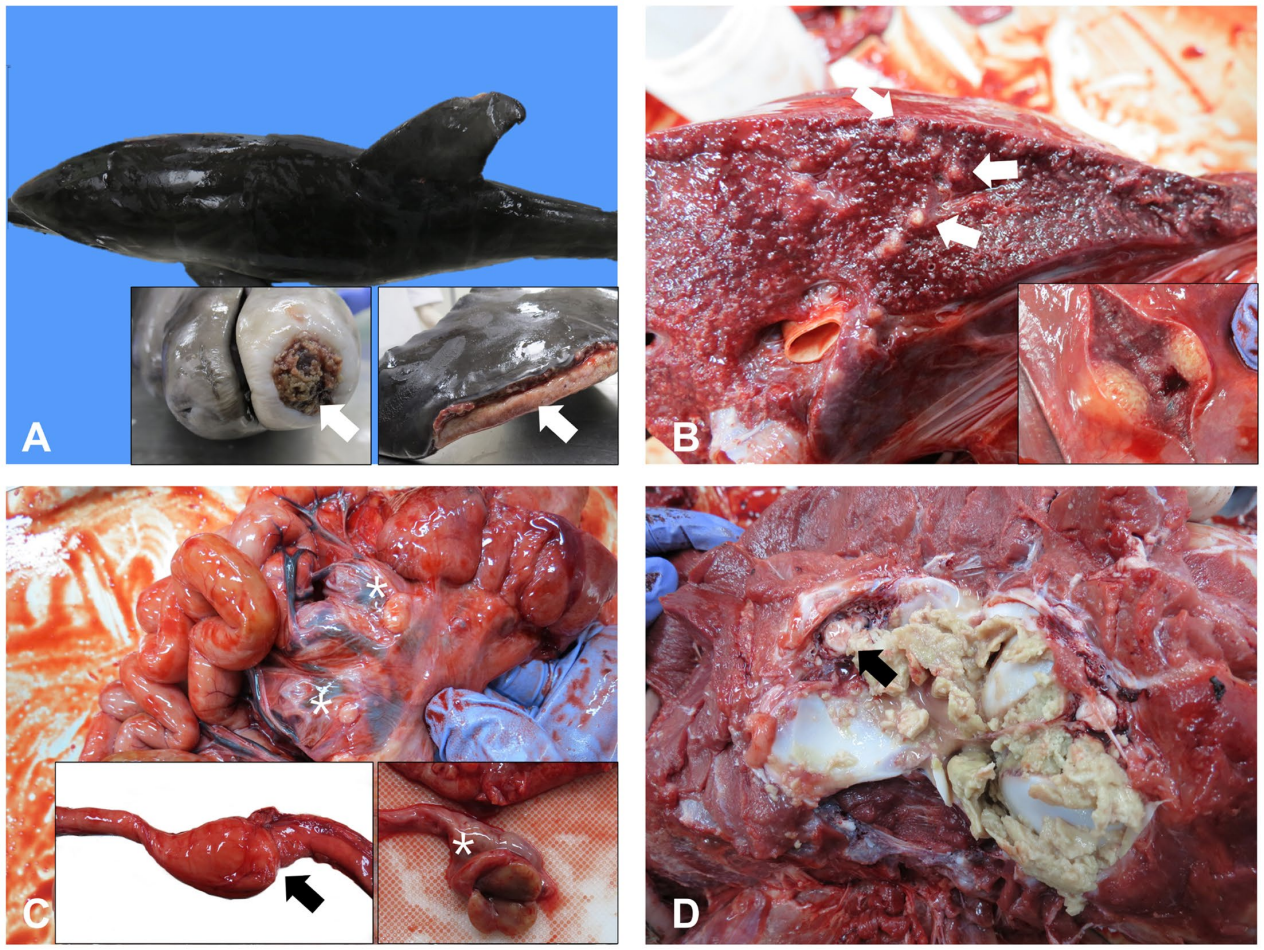
Representative gross lesions of a 25-day-old bottle nose dolphin. **(A)** Multiple cutaneous ulcerations and underlying inflammation and granulation tissue on the dorsal and pectoral fins and rostrum. **(B)** Multiple nodules on the lungs (arrows). **(C)** Enlargement and congestion of mesenteric lymph nodes (asterisks) and stricture and serosal hyperemia of the intestines (inset, arrow). **(D)** Fibrinopurulent arthritis in the atlantooccipital joint and extension of the fibrin in the spinal canal (arrow).

On gross examination, extensive cutaneous ulcerations were identified on the dorsal and pectoral fins and rostrum (Figure 1A). A large amount of blood-tinged ascites was found. Marked consolidation, congestion, and edema were observed in the whole lung tissue, along with generalized multifocal round soft yellow nodules between 5 and 10 mm in diameter (Figure 1B). The serosa of the intestinal tract was edematous and hyperemic (Figure 1C). In particular, middle part of the intestine was characterized by abnormal stricture with proximal dilation and congestion. Enlargement and congestion of mesenteric lymph nodes were also observed (Figure 1C). Consistent with the gastrointestinal tract findings, the liver and kidneys were swollen and congested. Although most organs showed signs of congestion or edema, the most significant findings at necropsy were seen in the cervical vertebral joint. Fibrinosuppurative arthritis in the atlantooccipital joint was observed (Figure 1D). The brain was edematous and swollen with flattened surface characterized by narrow sulci but widened gyri and congested.

**Figure 2:**
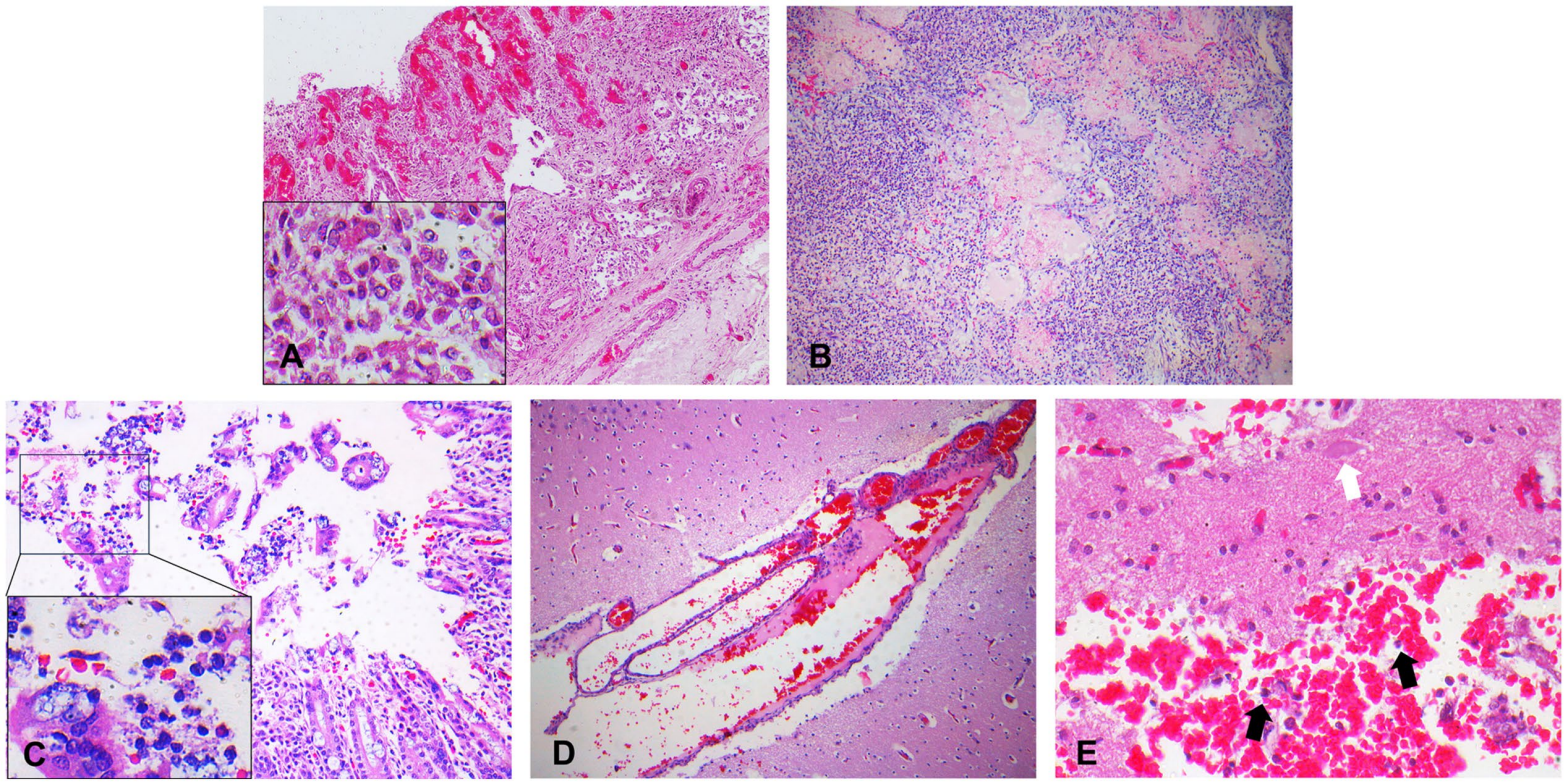
Representative photomicrographs of the hematoxylin and eosin (H&E) staining. **(A)** Trachea characterized by hemorrhage and necrosis of submucosal glands with infiltration of macrophages. 100x magnification, Inset: 800x magnification. **(B)** Lungs characterized by edema and congestion. Neutrophils and fibrin accumulation in the alveoli and larger airways. 100x magnification. **(C)** Intestines characterized by epithelial erosion, hemorrhages, detachement of epithelium and infiltration of the macrophages, lymphocytes, and neutrophils. 200x magnification, Inset: 800x magnification. **(D)** Brain characterized by meningeal vessel congestion. 200x magnification. **(E)** Intraparenchymal hemorrhages with neutrophils (black arrows) accompanied by neurodegeneration (white arrow). 400x magnification.

**Figure 3:**
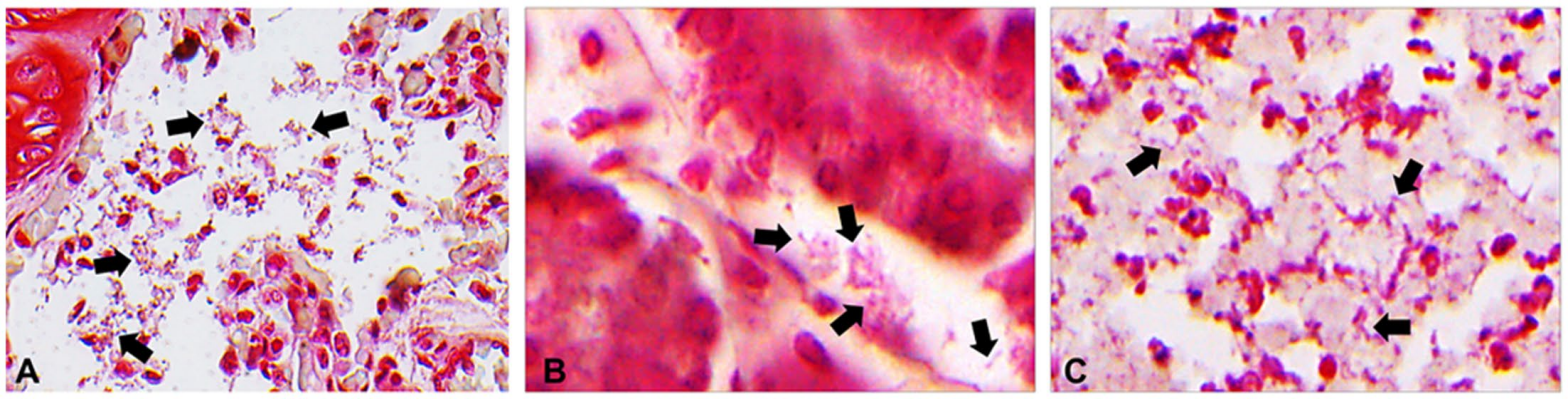
Representative photomicrographs of gram staining. **(A)** Gram-negative bacterial colonies (arrows) in the lungs. 800x magnification. **(B)** Gram-negative bacterial colonies (arrows) in the crypts of the intestines. 1,500x magnification. **(C)** Gram-negative bacterial colonies (arrows) in the fibrinosuppurative arthritis observed in the atlantooccipital joint. 1,500x magnification.

Microscopic examination revealed tracheitis, characterized by hemorrhage and necrosis of the submucosal glands accompanied by infiltration of inflammatory cells, such as macrophages (Figure 2A). Within the nodules, and to a lesser extent affecting all pulmonary parenchyma, are numerous neutrophils and fibrin accumulation in the alveoli and larger airways (Figure 2B). Epithelial erosion with hemorrhages, karyorrhectic debris of the enterocytes, lymphocytes and plasma cells with neutrophil and macrophage infiltration, and progressive destruction of epithelium were observed in the intestines (Figure 2C). Meningeal vessel congestion was observed in the brain (Figure 2D). Intraparenchymal hemorrhage with neutrophils, neurodegeneration such as loss of nuclei were observed in the spinal cord at the site of fibrinosuppurative arthritis (Figure 2E). Representative organs were further analyzed using Gram staining. Rod-shaped Gram-negative bacteria were present in the lungs, especially in the nodules, Gram-negative bacterial colonies were found inside the blood vessels, alveolar spaces, and interalveolar septum (Figure 3A). Bacterial colonies were also observed in the digestive system, including the liver and intestines. Bacterial colonies were observed in the crypt and the lumen of the intestines (Figure 3B). Aggregates of rod-shaped Gram-negative bacterial colonies, which had a morphology identical to that in other organs, were also observed in the fibrinosuppurative arthritis lesion in the atlantooccipital joint and the fibrin extended into the spinal canal (Figure 3C), suggesting bacteremia. Microbial culture revealed multiple infections with Gram-negative and Gram-positive bacteria, including *E. fergusonii*, *Shewanella haliotis*, *Enterococcus faecalis*, and *Staphylococcus schleiferi*, in the lung, liver, lymph nodes, blood samples from the jugular vein, and ascites from abdominal cavity (Table 1). Because *Escherichia fergusonii* was the only the bacterium identified in all the samples with characteristic pathologic lesions, it was thought to be the primary etiologic agent in this case.

**Table 1:** PCR-based bacterial identification of the isolates from a 25-day-old common bottlenose dolphin.

No.	Lung	Liver	Blood	Lymph nodes	Ascites
1	Escherichia fergusonii (**)	Escherichia fergusonii (**)	Escherichia fergusonii (**)	Escherichia fergusonii (**)	Escherichia fergusonii (**)
2	Enterococcus faecalis (*)	Enterococcus faecalis (*)	Enterococcus faecalis (*)		Enterococcus faecalis (*)
3	Staphylococcus schleiferi (*)		Staphylococcus schleiferi (*)		Staphylococcus schleiferi (*)
4	*Shewanella haliotis*				*Shewanella haliotis*

Isolated organisms were marked with the number of * following their frequency in the samples.

## Discussion

In the present case, a 25-day-old neonate dolphin showed several histopathological lesions indicating systemic bacterial infection on gross and microscopic examination. Enlargement of the mesenteric lymph nodes, multifocal nodules on the lungs, and fibrinosuppurative arthritis in the atlantooccipital joint with fibrin in the spinal canal were observed on gross examination. Hemorrhagic tracheitis, fibrinosuppurative bronchopneumonia, and enteritis, were the main microscopic findings. The gross and histopathological examinations strongly suggest that the animal succumbed to death due to bacterial sepsis. Mainly rod-shaped Gram-negative bacilli, were observed in several organs, including the trachea, liver, lungs, intestines, mesenteric lymph nodes, and fibrinosuppurative arthritis lesion observed in the atlantooccipital joint.

Culture and PCR analysis revealed a mixed infection of *E. fergusonii*, *S. haliotis*, *E. faecalis*, and *S. schleiferi* in the representative samples from this dolphin, but *E. fergusonii* was thought to be the primary causative agent because it was the only bacterium identified in all representative samples including blood sample collected from jugular vein which can rule out the postmortem contamination of the species. In the present case, several possible entry routes can be considered. One possible entry route of *E. fergusonii* into the body of this dolphin could have been transmural migration via the gastrointestinal tract. The intestines were characterized by necrosis of mucosal epithelium, accompanied by bacterial colonies in the lumens. The dolphin was only 25-day-old and might have been in an immunocompromised state owing to an immature immune system; therefore, opportunistic infection of *E. fergusonii* residing in the gastrointestinal tract cannot be excluded. It also explains the concurrent infection with other opportunistic pathogens such as *E. faecalis* and *S. schleiferi*. Another possible entry route could be via respiratory system. The dolphin was characterized by severe tracheitis and fibrinosuppurative pneumonia which were one of the main pathologic features in the present case. Considering *E. fergusonii* is easily found in the environment including water, respiratory system could be the main entry route. In that context, cutaneous abrasion lesions could not be excluded as well, since skin is the primary defensive barrier against pathogens and it could be another route of *E. fergusonii* entry. This hypothesis may explain the isolation of *S. haliotis*, which are abundant in water environment and can cause severe soft tissue infection via pre-existing cutaneous lesions (17), in lung and ascites of the dolphin.

Previous studies have suggested the clinical importance of *E. fergusonii* both in humans and terrestrial animals. Especially, in veterinary medicine, *E. fergusonii* has been reported to cause enteritis, and septicemia in horse and goat, fibrinonecrotic typhlitis in fowl, and fibrinous bronchopneumonia in cattle (18). Although a case report reported isolation of *E. fergusonii* in lungs of *Globicephala macrorhynchus* (19), its pathology and clinical importance have rarely been reported in aquatic organisms; therefore, it is not considered as the primary causative agent of sepsis in aquatic organisms, including dolphins. Prompt diagnosis and adequate clinical treatment are pivotal for the prognosis of sepsis in neonates. Antimicrobial resistance (AMR) was reported in *E. fergusonii* (19–21). Previous studies reported its wide-ranging resistance against nitrofurantoin, ciprofloxacin, ceftriaxone, amikacin, oxytetracycline, tetracycline, amoxicillin, amoxicillin-clavulanic acid, ampicillin, cefoxitin, penicillin, nalidixic acid, quinolone, and erythromycin (20, 21). Therefore, these antibiotics are not best option for the treatment of *E. fergusonii*. AMR against erythromycin for treatment of *Escherichia* species was reported in cetaceans (19). AMR against cephalothin, ampicillin, cefazolin were also observed in the bacteria isolated from cetaceans which might be ruled out for the treatment (19). In contrast, *E. fergusonii* are reported to be susceptible to florfenicol, gentamicin, and trimethoprim-sulfamethoxazole. Amoxicillin-clavulanic acid was also observed to be helpful for resolving bacteriuria (20, 21). In addition, gentamicin was found to be effective in treatment of a range of bacterial infection in cetaceans (19), so it can be the treatment option for *E. fergusonii* treatment in dolphins as well. However, antibiotic susceptibility and virulence test need to be performed to find the best option for the treatment which were the limitations of the present study. Thus, *E. fergusonii* infection and its virulence factors should be considered in captive environments. In this regard, we believe that this is the first known case of *E. fergusonii*-associated sepsis in a dolphin and can provide additional insights for the diagnosis of sepsis caused by this species in aquatic organisms, including dolphins.

## Data availability statement

The original contributions presented in the study are publicly available. This data can be found at: https://www.ncbi.nlm.nih.gov/nuccore/OR404993.

## Ethics statement

The dolphin was submitted for routine diagnostic post-mortem examination to the Department of Pathology and as a result not subject to animal ethics guidelines. The ownership of the dolphin belonged to the city, and the written informed consent for the participation was not required in accordance with the national legislation and the institutional requirements.

## Author contributions

S-MB was involved in data collection and interpretation and wrote the manuscript. S-WL, T-UK, JK, Y-JL, J-HY, and W-JK were involved in data collection. S-KC, JH, and K-SJ were involved in data analysis. J-KP supervised the entire process of data collection, analysis, and interpretation, reviewed, and revised the manuscript. All authors contributed to the article and approved the submitted version.

## Footnotes

^1^ http://www.ncbi.nlm.nih.gov/blast/

^2^ https://www.ncbi.nlm.nih.gov/nuccore/OR404993
